# Ventilation of intubated patients during HEMS hoisting operations

**DOI:** 10.1186/s13049-018-0508-z

**Published:** 2018-05-11

**Authors:** John Hollott

**Affiliations:** Hunter Retrieval Service, John Hunter Hospital, Lookout Rd, New Lambton Heights, NSW 2305 Australia

**Keywords:** Helicopters, Intratracheal intubation, Prehospital emergency care, Pulmonary ventilation, Rescue stretchers

## Abstract

In response to the review “Advanced airway management in hoist and longline operations in mountain HEMS - considerations in austere environments: a narrative review.” by Pietsch et al. we refer to recently published original research describing manual versus automatic ventilation of intubated patients during helicopter hoisting operations.

## Main text

The recently published “Advanced airway management in hoist and longline operations in mountain HEMS – considerations in austere environments: a narrative review.” [1] by Pietsch et al. is an excellent review of the issues involved in the helicopter hoisting of critically unwell patients, concentrating on alpine environments.

Contemporaneously to the authors' review we have published an original research paper “Ventilatory choices for intubated patients during helicopter stretcher winching” [2]. Concentrating on the method of ventilation, we compared the use of a self-inflating bag versus a mechanical ventilator during the helicopter hoisting (or winching) of a manikin, measuring airway pressures. The use of automatic ventilation was found to be more reliable, consistent and safer. As pointed out in Pietsch’s article, airway monitoring and disconnection is a major concern, but of a low risk if managed appropriately, and the safety benefits to the patient make it potentially the optimal method. In addition, the enhancement of situational awareness for the attendant when freed from the task of ventilation is likely to particularly benefit operational safety.

Our paper appears to be the only publication to date which has directly addressed the safety of ventilation during hoist operations. We believe that we have been able to demonstrate the advantages to both patient and team of utilising mechanical ventilation.

## Abbreviations

HEMS: Helicopter Emergency Medical Services
ICAR MEDCOM: International Commission for Mountain Emergency Medicine
